# Preliminary validation of the Chinese version of the Shame and Stigma Scale among patients with facial disfigurement from nasopharyngeal carcinoma

**DOI:** 10.1371/journal.pone.0279290

**Published:** 2022-12-22

**Authors:** Yuqi Cai, Yuan Zhang, Wangnan Cao, Fengsu Hou, Meiqi Xin, Vivian Yawei Guo, Yang Deng, Shenghao Wang, Xinyi You, Jinghua Li

**Affiliations:** 1 School of Public Health, Sun Yat-sen University, Guangzhou, Guangdong, China; 2 Department of Radiation Oncology, Sun Yat-sen University Cancer Center, State Key Laboratory of Oncology in South China, Collaborative Innovation Center for Cancer Medicine, Guangdong Key Laboratory of Nasopharyngeal Carcinoma Diagnosis and Therapy, Guangzhou, Guangdong, China; 3 Sun Yat-sen University Global Health Institute, School of Public Health and Institute of State Governance, Sun Yat-sen University, Guangzhou, Guangdong, China; 4 Department of Social Medicine and Health Education, School of Public Health, Peking University, China; 5 Department of Public Health, Shenzhen Kangning Hospital, Shenzhen, Guangdong, China; 6 Department of Rehabilitation Sciences, The Hong Kong Polytechnic University, Hong Kong, China; The University of Texas MD Anderson Cancer Center, UNITED STATES

## Abstract

**Objective:**

This study examined the reliability and validity of a Shame and Stigma Scale (SSS) and assessed shame and stigma among patients with facial disfigurement from nasopharyngeal carcinoma (NPC).

**Methods:**

Data were collected from 218 patients with NPC through a cross-sectional survey between January 14, 2020, and December 1, 2020. The original SSS is a 20-item scale with four dimensions (i.e., shame with appearance, sense of stigma, regret, and social/speech concern). We used Cronbach’s alpha and McDonald’s omega to assess reliability and exploratory factor analysis (EFA) to assess the factor structure. We also used Pearson correlation analysis to examine the relationship between each item and total score of scale items and convergent validity.

**Results:**

The final 18-item SSS had a Cronbach’s alpha coefficient of .89. The EFA revealed that the SSS has a four-factor structure: sense of stigma, social/speech concern, shame with appearance, and regret. These factors showed satisfactory reliability, with McDonald’s omega coefficients of .87, .77, .86, and .79, respectively. The scale showed significant relationship between each item and total score of scale items with respect to item–total correlations, item–subscale correlations, and item–other-subscale correlations. Convergent validity was supported by the significant positively correlated with the total scores for depression and anxiety.

**Conclusion:**

The SSS is valid and reliable in assessing shame and stigma and monitoring treatment compliance among patients with NPC.

## Introduction

Several studies have indicated that patients with nasopharyngeal carcinoma (NPC) have more psychiatric symptoms and poorer mental health than the general population [1]. Li found that in a study of 267 patients with NPC, the incidence rate for anxiety and depression were 35.2% and 25.5% respectively [2], which was higher than in the general population (4.4%) [3]. McDowell revealed that in a study of 107 patients with NPC aged 32 to 81, survivors of NPC experience many physical symptoms such as depression (25%), anxiety (37%), and fatigue (28%) [4]. It was reported that over 30% of cancer survivors had been subjected to negative attitudes and stereotypical views toward cancer, and approximately 10% of patients had experienced social discrimination due to cancer [5]. Given the severity of their poor mental health, patients with head and neck cancer (HNC) had higher risk of suicide than in the general population [6].

Shame is an affective state in which a sense of disgrace, dishonor, or humiliation may generate a desire to cover oneself or hide to escape [7]. Stigma is a state of social disapproval that results from a tarnished identity or disfigurement [8]. Previous studies have suggested that shame and stigma are significantly associated with psychological distress [9], poor mental health [10], passive disease coping behaviors, and poor social functioning [10] among patients with lung cancer [11] and breast cancer [9]. One meta-analysis showed that shame and stigma in patients with cancer were associated with poor mental health and other health outcomes such as increased depression (*z* value, .43, 95% confidence interval (CI), .32 to .54), increased anxiety (*z* value, .45, 95% CI, .23 to .68), severe impact on body image (*z* value, .64, 95% CI .29 to .99), and lower medical satisfaction (*z* value, -.33, 95% CI -.61 to -.06) [12]. Shame and stigma in patients with HNC are related to bodily disfigurement, isolation, and social resistance. Facial disfigurement is considered the single most stressful aspect of HNC [13]. Disfigurement has been associated with greater stigma, defined as shame, social isolation, fear of negative evaluation by others, or being the target of inappropriate behavior [14]. Further studies of cancer-related stigma are urgently needed.

There were some validated assessment tools of stigma and shame for lung cancer [15, 16] and breast cancer [17]. However, due to the complexity and different clinical features of different cancers, the assessment tool should be tailored accordingly. For example, stigma and shame scale for lung cancer has a specific factor to assess patients’ beliefs and stigma regards to smoking behaviors which is often associated with lung cancer [15]. While stigma in patients with breast cancer [18] and HNC patients [19] may be caused by altered body image. The Shame and Stigma Scale (SSS) developed by David W. Kissane is specific to patients with HNC. The item pool was identified from a review of the literature on shame and stigma in patients with bodily disfigurement and patients stigmatized by contagious diseases [19]. The scale has been translated and culturally adapted into a Portuguese version in Brazil and a Chinese version in Taiwan to measure shame and stigma among HNC patients.

There is very limited research on stigma among HNC patients and no researchers have yet investigated stigma associated with NPC. Local research on stigma is in its infancy. The SSS has shown good reliability in measuring the stigma of HNC patients. However, it is unclear whether it can be applied to NPC patients. It is also unclear if the Chinese version of SSS can be used to assess stigma in the Chinese population. This study aimed to validate the SSS in mainland China. We believe the results of this study will help to understand shame and stigma after treatment and to develop interventions that can improve mental health among patients with NPC.

## Materials and methods

### Study participants and recruitment

Ethical approval was obtained from the Ethics Committee of Sun Yat-sen University (No.2019-145). Data were collected through a cross-sectional survey conducted at Sun Yat-sen University Cancer Center (Guangzhou, China) between January 14, 2020, and December 1, 2020. The inclusion criteria for participants were: 1) aged 18 years or more; 2) documented diagnosis of NPC; and 3) no previous psychiatric disorders and other severe malignancies. The single exclusion criterion was inability to complete the questionnaire independently. Research assistants were recruited and trained to identify eligible patients. The research assistants provided study details to patients in the outpatient clinic and obtained written consent before the conduct of face-to-face interviews. Each interview lasted for 15–25 minutes. The quality of responses to the questionnaire was checked upon completion. We calculated the sample size according to the subject to item ratios of 10:1 [20]. There were 20 items in the original scale, thus the targeted sample size is 200. A total of 218 participants were included in the analysis.

### Descriptive statistics

Of the participants (*N* = 218), the age ranged from 20 to 74 (*M* = 47.34, *SD =* 11.39); 69.7% were male; 59.2% were older than 45 years; 56.0% lived in city; 89.9% were married; 55.5% had attended high school or higher; 47.7% had a job; 28.9% had an average monthly household income of RMB 3,001–5,000; 96.8% had medical insurance; 9.2% were diagnosed with NPC more than 5 years previously; and 63.3% had stage III NPC (Table 1).

**Table 1:** Demographic characteristics of individuals (*N* = 218).

Sociodemographic Variables	Response Categories	*n*	Percentage(*%*)
Age	≤45	89	40.8
	>45	129	59.2
Gender	Male	152	69.7
	Female	66	30.3
Ethnicity	Han Chinese	208	95.4
	Other ethnicity	5	2.3
	Unclear	5	2.3
Residence	Urban	122	56.0
	Rural	87	39.9
	Unclear	9	4.1
Civil status	Married	196	89.9
	Others (Single, divorced or widowed)	15	6.9
	Unclear	7	3.2
Primary caregiver	Spouse	155	71.1
	Children	35	16.1
	Parent and siblings	20	9.1
	Others	6	2.8
	Unclear	2	.9
Education	Junior high school and lower	95	43.6
	High school	54	24.8
	University and higher	67	30.7
	Unclear	2	.9
Occupational status	Full-time or part-time	63	28.9
	Retired	27	12.4
	Unemployed	79	36.2
	Others	41	18.8
	Unclear	8	3.7
Average monthly household income	Less than 3,000 RMB	56	25.7
	3,001–5,000 RMB	63	28.9
	5,001–10,000 RMB	56	25.7
	Above 10,000 RMB	33	15.1
	Unclear	10	4.6
Type of medical insurance	Urban employee-based medical insurance	77	35.3
	Urban resident-based medical insurance	26	11.9
	New cooperative medical scheme	63	28.9
	No medical insurance	4	1.8
	Others	45	20.7
	Unclear	3	1.4
Borrowing money to pay for treatment	Yes	57	26.1
	No	161	73.9
Time from first diagnosis	Less than 5 years	198	90.8
	More than 5 years	20	9.2
Clinical stage	I	1	0.5
	II	12	5.5
	III	138	63.3
	IV	67	30.7

### Measurements

#### The Chinese version of the SSS

The scale translation and adaptation procedures were as follows [21]: 1) a research panel of experts in NPC and psychology reviewed and discussed the item pool; 2) two translators translated the scale into Chinese; 3) we invited experts fluent in both Chinese and English to validate the translation and adapt it to the cultural context of China; 4) we did back translation and compare the back-translated versions of the scale with the original; 5) a draft version of the scale was pretested on 19 patients. The original version of the scale measured 4 dimensions, with 8 items (items 1–8) on shame with appearance, 6 items (items 9–14) on social avoidance, 3 items (items 15–17) on a sense of being avoided by others, and 3 items (items 18–20) on general regret at past behaviors. The items were scored from 0 (never) to 4 (always). Higher scores indicated more shame and stigma, which were anticipated in patients with NPC. The Cronbach’s alpha for the overall scale was .93 and the subscale alphas were .92 in shame with appearance, .89 in sense of stigma, .78 in regret, .78 in social/speech concerns respectively in an initial study [19].

#### The Chinese version of Generalized Anxiety Disorder 7-item scale (GAD-7)

This 7-item self-report scale was developed for screening symptoms of generalized anxiety in the primary care setting [22] and reflects the frequency of symptoms during a 2-week period [23]. Patients with GAD-7 scores of ≥ 10 are classified as at least “moderately ill” [23]. The possible range of scores is from 0 to 21, with a higher score indicating more severe levels of anxiety [23]. The Chinese version of GAD-7 has been shown to have good reliability and validity [24]. The Cronbach’s alpha was .94 in this study.

#### The Chinese version of Patient Health Questionnaire (PHQ-9)

This 9-item self-report version of the Primary Care Evaluation of Mental Disorders interview utilizes the Diagnostic and Statistical Manual of Mental Disorders fourth edition (DSM-IV) diagnostic criteria for the psychiatric diagnosis of major depression and has been well-validated in primary care and cancer populations [25, 26]. A score of ≥ 10 has good specificity for DSM-IV criteria for major depressive disorder. The possible range of scores is from 0 to 27, with higher scores indicating more severe levels of depression [25]. The PHQ-9 has been widely validated in many Chinese populations with good reliability and validity [27]. The Cronbach’s alpha was .91 in this study.

### Statistical analysis

All statistical analyses were conducted using IBM Statistical Package for the Social Sciences (SPSS) version 20.0 (IBM Corp., Armonk, NY, USA). The statistical significance level was a two-tailed value of *p* < .05. Means and standard deviations (*SD*) were presented for all items. The floor effect (at the very low end of the scale) and ceiling effect (at the very high end of the scale) were tested to assess if there is limitation in its responsiveness to clinical changes. An item was considered non-responsive if more than 70% of the responses were at the very low or very high end of the scale [28]. We used pearson correlation to determine item–total correlations, item–subscale correlations, and item–other-subscale correlations within the SSS. Cronbach’s alpha coefficients and McDonald’s omega were computed to measure the reliability related to internal consistency.

We used the Kaiser–Meyer–Olkin (KMO) and Bartlett’s sphericity tests to assess sampling adequacy and the appropriateness of the factor analysis. Exploratory factor analysis (EFA) was conducted to measure the factor structure. We used the principal component method for extraction and oblique rotation method. The initial criterion for retaining a factor was an eigenvalue higher than 1.0.

Pearson correlation coefficients were also derived between the SSS (total scores and subscale scores) and external variable (total scores of GAD-7 and PHQ-9) to assess the convergent validity of the SSS.

## Results

The mean total score for the SSS was 21.89 (*SD* = 12.08), with a range of 0 to 69. The percentages of very high scores ranged from .9% to 25.7%, while the percentages of very low scores ranged from 10.1% to 67.9%. There were no ceiling or floor effects. For the individual items, the mean score of item 7 (2.36) was the highest and the mean score of item 3 (.59) was the lowest (Table 2).

**Table 2:** Descriptive statistics for items of the Shame and Stigma Scale (*N* = 218).

Item Number	Score Range	*Mean*	*SD*	Floor Effect ^a^ (%)	Ceiling Effect ^b^ (%)	Skewness	Kurtosis	Weight (%)
1	0–4	1.65	1.308	24.8	10.1	.269	-1.075	4.45
2	0–4	.70	1.051	61.5	3.2	1.463	1.438	5.10
3	0–4	.59	1.004	67.9	2.3	1.690	2.084	5.16
4	0–4	2.24	1.381	14.7	25.7	-.191	-1.168	2.86
5	0–4	.70	.941	55.5	1.8	1.377	1.575	4.84
6	0–4	.68	.992	60.6	1.4	1.367	1.020	5.53
7	0–4	2.36	1.263	10.1	22.5	-.327	-.883	2.59
8	0–4	.74	.978	54.1	1.8	1.281	1.097	5.43
9	0–4	.83	1.161	56.4	4.1	1.298	.626	3.72
10	0–4	.82	.998	50.5	1.8	1.068	.498	5.51
11	0–4	.74	.970	55.0	1.4	1.188	.717	6.19
12	0–4	.75	.958	53.2	.9	1.160	.589	6.39
13	0–4	1.36	1.395	40.8	10.6	.574	-.995	4.80
14	0–4	.89	1.052	49.5	2.3	.975	.161	6.87
15	0–4	1.06	1.146	40.4	5.5	.985	.265	5.97
16	0–4	1.76	1.327	22.0	13.8	.241	-1.037	3.29
17	0–4	1.07	1.170	42.2	5.0	.903	-.051	6.45
18	0–4	.79	1.095	56.9	3.2	1.299	.809	6.82
19	0–4	.63	.962	63.3	1.4	1.421	1.251	6.49
20	0–4	1.97	1.404	17.9	22.5	.150	-1.224	1.56
Total Score	0–69	21.89	12.08	.5	.5	.812	.585	-

Note.

^a^ Floor effect: at the very low end of the scale.

^b^ Ceiling effect: at the very high end of the scale.

### Factor structures

#### Exploratory factor analysis

In EFA, the eigenvalues of all factors exceeded 1.0. We optimized the scale based on the result of EFA. Items 1 and 4 were deleted as they duplicated the meaning of item 3. This improved the overall value of Cronbach’s alpha from .873 to .886, so items 1 and 4 were excluded before final EFA. The KMO (.902) and Bartlett’s test (*χ*^2^ = 2028.174, *df* = 153, *p* < .0001) indicated good performance of the factor analysis. Four factors were derived for this scale, leaving 18 items in the final scale.

The four factors of the SSS for NPC were labeled as follows: 1) sense of stigma, 2) social/speech concern, 3) shame with appearance, and 4) regret. The eigenvalues of these factors were 7.50, 1.75, 1.38, 1.12, respectively, and explained 41.7%, 9.7%, 7.7%, 6.2% of the total variance (65.3%), respectively (Table 3).

**Table 3:** Exploratory factor analysis for the Shame and Stigma Scale (SSS).

Factor	Item	Factor loading			
		1	2	3	4
**Sense of stigma**	11. I feel ashamed for having developed cancer.	**.891**	.048	.015	-.020
	10. I am embarrassed when I tell people my diagnosis.	**.879**	-.042	.083	.003
	12. People avoid me because of my cancer.	**.824**	.130	-.056	.004
	14. I sense that others feel strained when around me.	**.669**	.098	-.047	.268
	9. I feel others consider me responsible for my cancer.	**.623**	-.314	-.096	-.059
	19. I avoid talking with others.	**.497**	.139	-.295	.156
	18. I am embarrassed by the change in my voice.	**.486**	.109	-.256	.271
**Social/Speech concern**	7. I enjoy going out in public (R).	.034	**.806**	-.032	-.016
	20. I am able to join conversations (R).	.015	**.773**	.022	-.058
**Shame with appearance**	3. I am ashamed of my appearance.	-.069	-.048	**-.904**	-.063
	2. I avoid looking at myself in the mirror.	-.137	.100	**-.798**	.106
	6. I avoid meeting people because of my looks.	.103	.104	**-.778**	-.103
	8. I am distressed by the changes in my face or neck.	.144	-.123	**-.688**	-.016
	5. I feel people stare at me.	.160	-.167	**-.479**	.172
**Regret**	16. I would do many things differently if given a second chance.	-.149	-.098	.089	**.927**
	15. I have a strong feeling of regret.	.203	.062	-.003	**.750**
	17. I feel sorry about things I have done in the past.	.126	.002	-.272	**.635**
	13. I have an urge to keep my cancer a secret.	.313	-.068	-.066	**.418**
Cronbach’s alpha		.90	.51	.82	.79
McDonald’s omega		.87	.77	.86	.79
Initial eigenvalues		7.50	1.75	1.38	1.12
Cumulative % of variance explained		41.7	9.7	7.7	6.2

Note.

The extraction method was principal component analysis. The rotation method was Direct Oblimin. Factor loadings above .40 are in bold. Reverse-scored items are denoted with an (R).

#### Item analysis and internal consistency

The item–total correlation coefficients ranged from .081 to .792 (Table 4). The item–subscale correlation coefficients ranged from .669 to .843 (all *p* < .01, Table 4). The item–other-subscale correlation coefficients ranged from -.184 to .626 (Table 4). All item–subscale correlation coefficients were higher than the correlation coefficients between the same item and other subscales. The Cronbach’s alpha coefficient was .89 and ranged from .51 to .90 for the four subscales. The McDonald’s omega coefficient was .78 and ranged from .77 to .87 for the four subscales (Table 3).

**Table 4:** Item analysis for the Shame and Stigma Scale.

**Item number**	**Cronbach’s alpha if item is deleted**		**Item–total correlation**	**Item–subscale correlation**	**Item–other–subscale correlation**
	**Subscale**	**Total**			
2	.791	.879	.592**	.770**	.412**, .089, .362**
3	.772	.879	.594**	.805**	.460**, -.012, .321**
5	.818	.879	.610**	.681**	.519**, -.115, .459**
6	.774	.878	.639**	.798**	.498**, .132, .328**
7	-	.898	.149**	.799**	.031, .033, -.077
8	.785	.878	.640**	.772**	.530**, -.057, .412**
9	.910	.881	.568**	.669**	-.184**, .407**, .423**
10	.886	.876	.690**	.795**	-.036, .434**, .492**
11	.879	.875	.733**	.843**	.010, .477**, .483**
12	.880	.874	.760**	.837**	.090, .514**, .490**
13	.789	.879	.627**	.750**	.522**, -.083, .408**
14	.881	.872	.785**	.833**	-.001, .514**, .620**
15	.695	.875	.694**	.835**	.595**, -.058, .401**
16	.760	.887	.446**	.770**	.318**, -.193**, .202**
17	.718	.873	.741**	.805**	.626**, -.098, .552**
18	.883	.872	.792**	.820**	.025, .583**, .581**
19	.887	.874	.755**	.783**	.058, .582**, .505**
20	-	.903	.081	.841**	-.047, -.010, -.148*

Note.

**p < .01

*p < .05.

Item–total correlations: Pearson correlation coefficient between each item and the overall scale (i.e., the total scores of the 18 items). Item–subscale correlation: Pearson correlation coefficient between each item and its corresponding subscale. Item–other–subscale correlation: Pearson correlation coefficient between each item and the other subscale.

#### Convergent validity

The total scores from the SSS were positively correlated with the total scores for depression (*r* = .507, *p* < .01) and anxiety (*r* = .475, *p* < .01). Significant positive correlations were also found between scores for three subscales (sense of stigma, shame with appearance, regret) of the SSS and scores for depression (*r* = .328-.530, *p* < .01) and anxiety (*r* = .290-.506, *p* < .01) (Table 5).

**Table 5:** Pearson correlation among the Shame and Stigma Scale, PHQ and GAD-7.

	Depression Scores (SUMPHQ)	Anxiety Scores (SUMGAD)
Shame and Stigma Scale	.507**	.475**
Subscale 1: Sense of stigma	.530**	.506**
Subscale 2: Social/Speech concern	.003	.020
Subscale 3: Shame with appearance	.417**	.386**
Subscale 4: Regret	.328**	.290**

Note.

**p < .01.

SUMPHQ = Sum of Patient Health Questionnaire. SUMGAD = Sum of Generalized Anxiety Disorder 7-item Scale.

## Discussion

This is the first study in mainland China to validate the SSS among a sample of NPC patients. This study adapted the SSS in a Chinese sample with good reliability and structural validity and was tailored to the clinical features of NPC. The scale may have the potential to be widely used to assess stigma and shame among Chinese NPC patients in the future psychosocial researches. The results of this study will help to understand shame and stigma after treatment and to develop interventions that can improve mental health among patients with NPC. The internal consistency measured by Cronbach’s alpha coefficient was .89 and ranged from .51 to .90 for the four subscales. The McDonald’s omega was .78 and ranged from .77 to .87 for the four subscales. The convergent validity of the SSS was supported by its significant correlations with depression and anxiety. We did not see any differences in stigma among NPC patients with different demographic characteristics.

The SSS showed satisfactory internal consistency. The Cronbach’s alpha in our study was .89, which is in the optimal range for internal consistency and consistent with the original scale from Kissane (Cronbach’s alpha = .93) [19]. Kissane’s study was based on a sample of patients with squamous cell carcinoma of the oral cavity, which is different from NPC. In our study, items 1 and 4 were deleted to improve the internal consistency of the scale before factor analysis. Both items had reversed wording and it has been reported that reversed items may weaken scale validity [29]. The remaining items were grouped into four dimensions by factor analysis, including factor 1 (sense of stigma), factor 2 (social/speech concern), factor 3 (shame with appearance), and factor 4 (regret). Based on the result of the EFA, item 7 (“I enjoy going out in public”) was assigned to the social/speech concern subscale in this study rather than to shame with appearance as in the original scale. Considering the results of EFA and the meaning of item 7, it may be more appropriate to classify it into the subscale of social/speech concern. Convergent validity was confirmed by the positive correlations between SSS scores and scores from depression and anxiety scales. This is consistent with findings from previous studies [19]. We found good internal consistency for the SSS. The correlations show that visible disfigurement including scarring, hair loss, sunburn, and other facial disfigurements due to radio-chemotherapy among NPC patients can lead to low self-esteem, loss of self-confidence, regret of treatment, and shame and stigma. These result in negative psychological impacts such as depression, anxiety, and social avoidance [30–33].

We acknowledge several limitations. First, due to the small sample size, we did not conduct confirmatory factor analysis to verify the structure of the SSS and we encourage future studies to apply the scale and verify its factor structure in China. Second, we only recruited participants from one hospital in Guangzhou. The sample may not represent the general population appropriately due to differences in demographic characteristics and clinical severity. And we expect studies would apply the SSS with a more representative sample of Chinese population. Furthermore, this research was primarily focused on the structural validity of the Chinese version of SSS, predictive validity or concurrent validity were not assessed due to lack of gold standard. Finally, the scale lacked sufficient relationship between the total scale and the subscale of social concern. Though its low correlations with total and other subscales (< .3) may suggest potential removal [34], we kept it in the final scale considering that the potential domain-relevant contributions of the items may supersede the absolute correlation values [35]. Given the limitations, the results should be interpreted with caution. Nevertheless, this study carries several implications for further research and current practice.

## Conclusions

Our preliminary evidence suggested that the SSS for NPC is valid and reliable, and can provide a reasonable assessment of shame and stigma in NPC patients. However, its application will require additional modifications and validation efforts among more representative samples. Further research is warranted to modify and test the present SSS in a larger and more diverse sample of patients with NPC.

## Supporting information

S1 Table The Chinese version of Shame and Stigma Scale. (DOCX)
