# Supplementary material for: Preliminary validation of the Chinese version of the Shame and Stigma Scale among patients with facial disfigurement from nasopharyngeal carcinoma
Source: PLoS One. 2022 Dec 22;17(12):e0279290. doi: 10.1371/journal.pone.0279290 (PMC9778931; doi:10.1371/journal.pone.0279290)
Supplement: S1 Table — (DOCX) [file pone.0279290.s001.docx]

**Supplementary Table.** The Chinese version of Shame and Stigma Scale

**鼻咽癌患者病耻感与污名化中文量表**

| **过去1周内，根据您的真实感受，在后面的空格里面打对勾，“√”。** | 从不 | 偶尔 | 有时 | 经常 | 一直 |
| --- | --- | --- | --- | --- | --- |
| 1. 我喜欢我的模样* | 0 | 1 | 2 | 3 | 4 |
| 1. 我避免在镜子里看到自己 | 0 | 1 | 2 | 3 | 4 |
| 1. 我为我的外貌感到羞耻 | 0 | 1 | 2 | 3 | 4 |
| 1. 我对我的脸或脖子的外观很满意* | 0 | 1 | 2 | 3 | 4 |
| 1. 我觉得人们总盯着我看 | 0 | 1 | 2 | 3 | 4 |
| 1. 因为我的外观，我不喜欢见人 | 0 | 1 | 2 | 3 | 4 |
| 1. 我喜欢出现在公共场合* | 0 | 1 | 2 | 3 | 4 |
| 1. 我的脸或脖子的变化让我感到很痛苦 | 0 | 1 | 2 | 3 | 4 |
| 1. 我觉得别人认为我应该为我的癌症负责 | 0 | 1 | 2 | 3 | 4 |
| 1. 当我告诉他人我的诊断时，我感到尴尬 | 0 | 1 | 2 | 3 | 4 |
| 1. 我为患癌症感到羞愧 | 0 | 1 | 2 | 3 | 4 |
| 1. 人们因为我的癌症而避开我 | 0 | 1 | 2 | 3 | 4 |
| 1. 我渴望将自己患肿瘤的事情保密 | 0 | 1 | 2 | 3 | 4 |
| 1. 我感觉别人在我身边时他们都会感到压力 | 0 | 1 | 2 | 3 | 4 |
| 1. 我有强烈的遗憾感 | 0 | 1 | 2 | 3 | 4 |
| 1. 如果给我第一次机会，我会做很多事情 | 0 | 1 | 2 | 3 | 4 |
| 1. 对我过去所做的事情感到愧疚 | 0 | 1 | 2 | 3 | 4 |
| 1. 我声音变化让我感到尴尬 | 0 | 1 | 2 | 3 | 4 |
| 1. 我避免与他人交流 | 0 | 1 | 2 | 3 | 4 |
| 1. 我可以加入对话* | 0 | 1 | 2 | 3 | 4 |
